# The effects of social comparison and depressive mood on adolescent social decision-making

**DOI:** 10.1186/s12888-020-02928-y

**Published:** 2021-01-05

**Authors:** Yixin Hu, Mengmeng Zhou, Yunru Shao, Jing Wei, Zhenying Li, Shike Xu, Phil Maguire, Dawei Wang

**Affiliations:** 1 School of Psychology, Shandong Normal University, Jinan, China; 2 Department of Statistics, University of Connecticut, Mansfield, USA; 3 Department of Computer Science, National University of Ireland, Galway, Ireland

**Keywords:** Social decision-making, Ultimatum game, Depressive mood, Social comparison, Gain context and loss context, Adolescents

## Abstract

**Background:**

Based on social comparison theory, two experiments were conducted to explore the effects of depression and social comparison on adolescents, using the ultimatum game (UG).

**Methods:**

Before the formal experiment began, a preliminary experiment tested the effectiveness of social comparison settings. This study used the UG paradigm to explore adolescents’ social decision-making in the context of gain and loss through two experiments. These experiments were designed as a 2 (group: depressive mood group, normal mood group) × 2 (social comparison: upward, downward) × 3 (fairness level: fair 5:5, unfair 3:7, extremely unfair 1:9) three-factor hybrid study.

**Results:**

(1) The fairer the proposal was, the higher the sense of fairness participants felt, and the higher their acceptance rate. (2) The acceptance rate of the participants for downward social comparison was significantly higher than that for upward social comparison, but there was no difference in fairness perception between the two social comparisons. (3) Under the context of gain, the acceptance rate of the depressive mood group was higher than that of the normal mood group, but there was no difference in the acceptance rate between the depressive mood group and the normal mood group under the loss context. Depressive mood participants had more feelings of unfairness in the contexts of both gain and loss. (4) The effects of depressive mood, social comparison and the fairness level of distribution on social decision-making interact.

**Conclusions:**

The interaction of social comparison, depressive mood and proposal type demonstrates that besides one’s emotion, cognitive biases and social factors can also have an effect on social decision-making. These findings indicate that behavioral decision boosting may provide an avenue for appropriate interventions in helping to guide adolescents to make social decisions.

## Background

Social decision-making, including a variety of phenomena such as reciprocity, cooperation, fairness perceptions and fairness judgment, refers to decision-making behavior in the context of social interaction [30, 36]. For humans living in a complex social environment, it is during social interaction that many crucial decisions are made [8]. Adolescence is a developmental period characterized by dramatic changes in both physiology and psychology, and it is a time when individuals are engaged in more social communication and faced with more social decisions [51, 78]. For instance, Zhang, Xu and Ding [79] demonstrated that the cooperative behavior of adolescents appeared to decline with increase in age. Although adolescents could perform similarly to adults in cognitive decision abilities [53], the unique characteristics of the social decision-making of adolescents are determined by their emotional sensitivity, instability, and poor self-control [4]. Therefore, exploring the characteristics, and capturing the rules of social decision-making in adolescents could serve to improve their social decision-making ability and promote scientific psychosocial education.

The Ultimatum Game (UG) is a standard paradigm used to explore social decision-making, in which the behavioral response of participants toward different levels of unfairness can be investigated by simulating the allocation of funds in social interaction [31, 37]. Typical UG involves a “Proposer” and a “Responder”, and a certain sum of money is shared by both at the beginning of each round. The proposer then proposes a share of this money to the responder, who can either accept or reject this proposal. If the responder accepts the proposal, money is shared in proportion to the proposal; however, if the responder refuses, both players gain nothing in that round. According to the principle of benefit maximization in game theory, responders should accept any nonzero proposals; rejecting an unfair proposal would be considered irrational from this economic perspective [11]. Nevertheless, studies indicate that unfair proposals are often rejected [14, 75, 81]. Thus, there are two potential outcomes of decision-making for responders—to accept or to refuse. While making decisions, the responder must judge the acceptability of the proposal according to her or his own fairness standards [44]. Therefore, indicators such as the acceptance rate of proposals and fairness perceptions are usually used to investigate the decision mode of the responder [32, 82].

Adolescents experience dramatic changes in both physiology and psychology [51, 78]. As a result, social decision-making in adolescents has attracted the attention of researchers [49]. This is a vulnerable period when the occurrence rate of depression increases dramatically [48], with the detection rate of depression ranging from 20 to 44% [26]. Depression and decision-making are closely connected [21]. Relative to healthy people, those with depression perform somewhat maladaptively in terms of fairness, cooperation, altruism and other social principles (e.g., decrease in cooperation, excessive altruism). As such, the acceptance rate of unfair allocation appears to be higher in individuals with depression, suggesting that they attempt to maintain “group fairness” at the expense of self-interest. However, Scheele et al. [55] suggested that individuals with depression are more likely to reject unfair proposals and tend to regard others’ proposals as unfair ones. The inconsistent conclusions reported by studies of decision-making in individuals with depression may be due to the different levels of depression they are experiencing [68] or the consumption of different psychotropic drugs [19]. Indeed, the significant relationship between pubertal transition and depressive mood that does not meet the diagnostic criteria for depression has also been emphasized in several studies [27, 28, 38]. Specifically, depressive mood was found to increase linearly for 10–19 year-olds, presenting as a persistent symptom of dispiritedness [66]. This raises the question of whether adolescents with depressive mood show differences in social decision-making from their normal mood peers. Based on the available evidence, we propose that adolescents with depressive mood may manifest higher acceptance rates of unfair allocation than their healthy counterparts, while their fairness perceptions may be lower. These proposals are formalized in terms of the following hypotheses.

- H1: Depressive mood has a significant effect on the social decision-making of adolescents. Individuals with depressive mood tend to accept unfair allocation and develop fewer fairness perceptions compared to those who are healthy.

A prominent change in adolescents’ evaluation of fairness, trust and reciprocity might alter their considerations of fairness, which would then have an effect on their social decision-making [16]. In line with fairness theory, individuals consider whether their costs and benefits are basically the same in comparison with others; hence, responders care not only about their own benefit but also about proposers’ relative gains. This suggests that different perceptions of fairness for different allocation proportions may lead to different social decision-making behaviors.

Allocation proposals were categorized inconsistently in previous studies using UG. According to the proportion that responders and proposers gained, proposals could be categorized into two levels. For example, Wu and Zhou [70] categorized proposals into fair (5:5, 4:6) and unfair (2:8, 1:9) levels; Destoop et al. [19] categorized proposals into fair (5:5) and unfair (3:7, 2:8 and 1:9) levels; and Gradin et al. [29] categorized proposals into fair (38–50%) and unfair (8–33%) levels according to the percentage of the total money that responders gained. Meanwhile, some researchers set three levels of proposals. For example, Huang et al. [39] categorized proposals into fair (5: 5), generous (9:1, 8:2) and selfish (1:9, 2: 8) levels; Wang et al. [68] categorized proposals into fair (50–40%), unfair (33–25%) and extremely unfair (20–10%) levels. Other researchers have set five levels of proposals according to the specific amount of money [64, 83]. As we can see, most of the studies set two, three or five levels of allocation proposals based on the levels of fairness. However, it is too complicated to compare experiments using previous sets because of the one-to-many relationship, which means that there are multiple allocations in one level. Thus, in the current study, to better explore the effect of fairness perception differences caused by different proposals on the social decision-making of adolescents, the allocation proposals were categorized into three levels (fair (5:5), unfair (3:7) and extremely unfair (1:9)), which would not only simplify the proposers’ choices and the experiments, but also increase the representativeness of the proposals for different levels of fairness. Hypothesis 2 was posited based on this notion:

- H2: Fairness level has a significant effect on the social decision-making of adolescents. With the unfair level of proposals increasing, responders are more likely to reject the proposal and perceive it unfairly.

Decision-making is more likely to be influenced by social comparison in the context of social interaction [54]. Festinger [24] proposed that, following typical social comparison theory, individuals could not help but make comparisons with others to learn about themselves, and the results of such comparisons could have an effect on their own decision-making behavior. For adolescents studying in a collective environment and interacting with peers frequently, their behavior and decision-making would be particularly affected by social comparison [43]. Social comparison is concerned with the processes involved in comparing the position and status (including ability, social status, behavioral pattern) of oneself with others [58, 72]. However, it is the tendency toward social comparison [41, 81], in particular, its frequency, that came to the attention of researchers, with less attention given to the different ways in which comparisons are made. From the perspective of social comparison, there are three types: lateral comparison, downward comparison and upward comparison. Lateral comparison is comparing oneself with those who are similar, while downward and upward comparison refer to comparing oneself with those who are, respectively, weaker in some respects, or better off. The different types of social comparison certainly have an impact on individuals’ social decision-making [62, 74]. Specifically, Brickman et al. [7] demonstrated that upward comparison might hurt an individual’s self-esteem and make her or him perceive threats and experience negative emotions elicited by this comparison, which may lead to a higher rate of rejection toward unfair allocation proposals. Additionally, it was found that individuals’ self-esteem and happiness could be raised by downward comparison, which may also moderate their negative emotions when facing unfair allocations and thereby increase their rate of acceptance [69]. Given the strength of the evidence, it is certain that the type of social comparison has a noteworthy impact on social decision-making. Despite previous studies focused on one aspect of social comparison, little is known about the effect of social comparison on social decision-making. Therefore, we intended to comprehensively explore the effect of upwards and downwards social comparison on social decision-making. Hypothesis 3 was proposed with this aim in mind:

- H3: Social comparison has a significant effect on the social decision-making of adolescents. Compared to individuals who use upward comparisons, those who use downward comparisons are more likely to accept proposals and develop perceptions of fairness.

In light of their development of cognitive ability and increase in social communication, adolescents may make more social decisions that are significantly affected by social comparison, depressive mood and levels of fairness of proposals. Studies have shown that social comparison is related to depression and further indicated that depressive individuals compare themselves with others more frequently and use more upward comparisons [1]. Additionally, social comparison is also under the effect of fairness level in UG; that is, unfair proposals could trigger more downwards comparisons to allow individuals to maintain a position of relatively higher self-esteem [46].

Thus, we reasoned that the effect of social comparison, depressive mood, and levels of fairness of proposals on social decision-making would be interactive and complex, rather than completely independent.

- H4: The interaction of fairness level, social comparison and depressive mood is significant in the process of social decision-making among adolescents. Fairness level, social comparison, and depressive mood have a common effect on the social decision-making of adolescents.

Social decision-making usually contains two situations—gain or loss—with individuals valuing the trade-off of loss more than gain [32]. The framing effect on social preferences proposes that individuals are influenced by the framework of optional proposals when making decisions, which then alters their tendency toward cooperation, reciprocity and altruism [18]. Previous studies using UG have revealed that, for responders, comparing their profits to that of proposers could influence their perceptions of fairness in the situation of gain, whereas the impact of loss on their perceptions of fairness remains unclear [44]. With a typical UG, there is no difference between decisions in the gain situation, whereas the proposer and the responder both share the loss in the loss situation. If the proposal is accepted, the money is divided between both players according to the proposal (i.e., they experience the gain together according to the proportion in the proposal). By contrast, both players split the loss of the total amount if the proposal is rejected. It was found that individuals show lower fairness perceptions and a higher rejection rate of unfair proposals in the condition of loss [32]. However, Li et al. [44] demonstrated that the acceptance rate is higher in loss situations than in gain situations. In summary, studies of social decision-making in different contexts have not reached a consensus, suggesting that further clarification is required. Accordingly, in the following experiments we explore the social decision-making of adolescents in gain and loss situations.

Taken together, starting with multiple variables and using the UG, we examine the effect and interaction mechanism of fairness level, social comparison and depressive mood on the social decision-making of adolescents in different situations. First, experiment 1 examines the effect of depressive mood and social comparison on the social decision-making of adolescents in gain situations. Subsequently, experiment 2 explores the effect of depressive mood and social comparison on the social decision-making of adolescents in a loss situation.

## Methods

### Experiment 1: the effect of depressive mood and social comparison on social decision-making of adolescents in a gain situation

#### Participants

A total of 216 participants (84 males) with a mean age of 15.27 (SD = 1.36) were selected using cluster random sampling from a high school. The 13-items Beck Depression Inventory (BDI-13), Self-rating Depression Scale (SDS), and Self-rating Anxiety Scale (SAS) were used in a collective evaluation to screen individuals with depressive mood, and to rule out the effects of anxiety. Standards for the different groups are as follows:

##### Depressive mood group

The following standards were met at the same time: (1) the score on BDI-13 being higher than 4; (2) the score on SDS being higher than 53 (including 53); and (3) the score on SAS being less than 50. Normal mood group: the following standards were met at the same time: (1) the score on BDI-13 being less than 4 (including 4); (2) the score on SDS being less than 53; and (3) the score on SAS being less than 50.

Students who volunteered to take part in this experiment were selected, and the three scales were used again before the experiments in case of a change in their mood. Eventually, 36 normal mood students (15 males), with an average age of 16.36 (SD = 0.64), and 40 depressive mood group students (18 males), with an average age of 16.38 (SD = 0.59), were recruited for the experiment (normal mood: BDI-13 (1.17 ± 1.44), range = 4, from 0 to 4, SAS (33.58 ± 7.38), range = 23, from 25 to 48, SDS (38.19 ± 11.74), range = 28, from 25 to 53; depressive mood: BDI-13 (7.45 ± 2.57), range = 11, from 5 to 16, SAS (44.00 ± 3.72), range = 15, from 34 to 49, SDS (58.19 ± 4.39), range = 19, from 54 to 73). We conducted a power analysis using G*power following prior studies [22, 55], with the result showing that 1-β = 0.9 (effect size = 0.20, alpha = 0.05, total sample size = 76, number of groups = 2, number of measurements = 6, corr among rep measures = 0.5, nonsphericity correction ε = 1).

#### Measurements

##### 13-items Beck depression inventory (BDI-13)

The 13-item BDI was originally developed by Beck and Beamesderfer [2] to assess the severity of depression. Each item is rated on a four-point scale (ranging from 0 to 3), reflecting the degree of depression symptoms. The total score was used as a main statistical indicator according to the following standards: a total score ≤ 4 was classified as no or mild depression, 5–13 as mild depression, 14–20 as moderate depression, and ≥ 21 as major depression. The BDI-13 showed adequate reliability with a Cronbach’s alpha of 0.97 in the current study. Moreover, adequate reliability and validity has been demonstrated in prior studies, indicating that the BDI-13 is an effective measurement for assessing depression [67, 73, 80].

##### Self-rating depression scale (SDS)

The SDS was originally developed by Zung [85] to assess the severity of depression and changes during treatment. The scale has since been revised by Chinese researchers [17]. There are 20 items in the SDS, each of which is scored on a scale of 1–4. The main statistical indicator is the total score, and the standard score ranges from 25 to 100 after conversion. Twenty questions assessed depression according to the following standards: A standard score of 53 was classified as a critical value, 53–62 as mild depression, 63–72 as moderate depression, and ≥ 72 as major depression. The Cronbach’s alpha of SDS in the current study was 0.90.

##### Self-rating anxiety scale (SAS)

The SAS is a 20-item self-report scale developed by Zung [84] and revised by Chinese researchers to evaluate the subjective feelings of anxious individuals [17]. Each item is rated on a four-point scale, and the main statistical indicator is the total score. After conversion, the higher the standard score, the more severe the symptoms. A standard score < 50 was classified as healthy, 50–59 as mild anxiety, 60–69 as moderate anxiety, and ≥ 70 as major anxiety. The Cronbach’s alpha of SAS in the current study was 0.94.

#### Design

A three-factor mixed design of 2 (group: depressive mood group, normal mood group) × 2 (social comparison: upward, downward) × 3 (fairness level: fair 5:5, unfair 3:7, extremely unfair 1:9) was used. The independent variables included group, social comparison and fairness level. Group was a between-subject variable, while social comparison and fairness level were within-subject variables. The dependent variables were acceptance rates and fairness perceptions of allocation proposals. Specifically, the acceptance rate referred to the proportion of times participants chose to accept as a proportion of the total number of gain situations. Statistical analyses were performed using SPSS 16.0. We employed Benjamini and Hochberg’s Method to control the family wise error rate. The analyses showed that *p* < 0.053, indicating that the results were significant [57].

#### Procedure

First, BDI-13, SDS and SAS were used to select participants who met the criteria for inclusion. Subsequently, participants performed the formal experiment one by one by appointment. The typical UG was used to evaluate the acceptance rate and fairness perceptions toward allocation proposals. Upon arrival, each participant was informed as to the rules of the game as follows: “You and Xiaoli, who is a virtual stranger, will work together on this task, in which 100 yuan will be allocated. The allocation proposals will be proposed by Xiaoli”. Participants were informed that Xiaoli shared the same gender and age as themselves. “You have the right to accept or reject his/her offer. If the offer is accepted, then the money will be shared by both of you according to the offer. On the other hand, if the offer is refused, both of you will gain nothing. Your decision in each round will be kept confidential.” In addition, participants were informed of the amount of money gained in each allocation by a randomly selected group of seven students (upward social comparison group: the amount of money gained by seven students in group A was on average 10 yuan more than that of the participant; downward social comparison group: the amount of money gained by seven students in group B was on average 10 yuan less than that of the participant). Each trial was independent, so that that later games would not be influenced. The total profit was the sum of the profits (tokens) of each round. Gifts were gel pens (the best), ink pen refills, and bookmarks (the worst), one of which would be received by participants according to their total profits. The more profits participants gained, the better the gifts they would get. Participants could proceed to the practice phase when they fully understood the instructions.

The procedure of the experiment was written using E-prime, using computers to present the experimental stimulus. One practice block and six experimental blocks were contained in the experiment. Practice block: The same experimental procedure and content were included in the practice block and experimental blocks. The requirements and procedures of the full experiment were introduced to participants who were then told to practice for 5 trials. Participants proceeded to experimental blocks when they had mastered the procedures and content of the experiment.

##### Experiment blocks

**Fig. 1:**
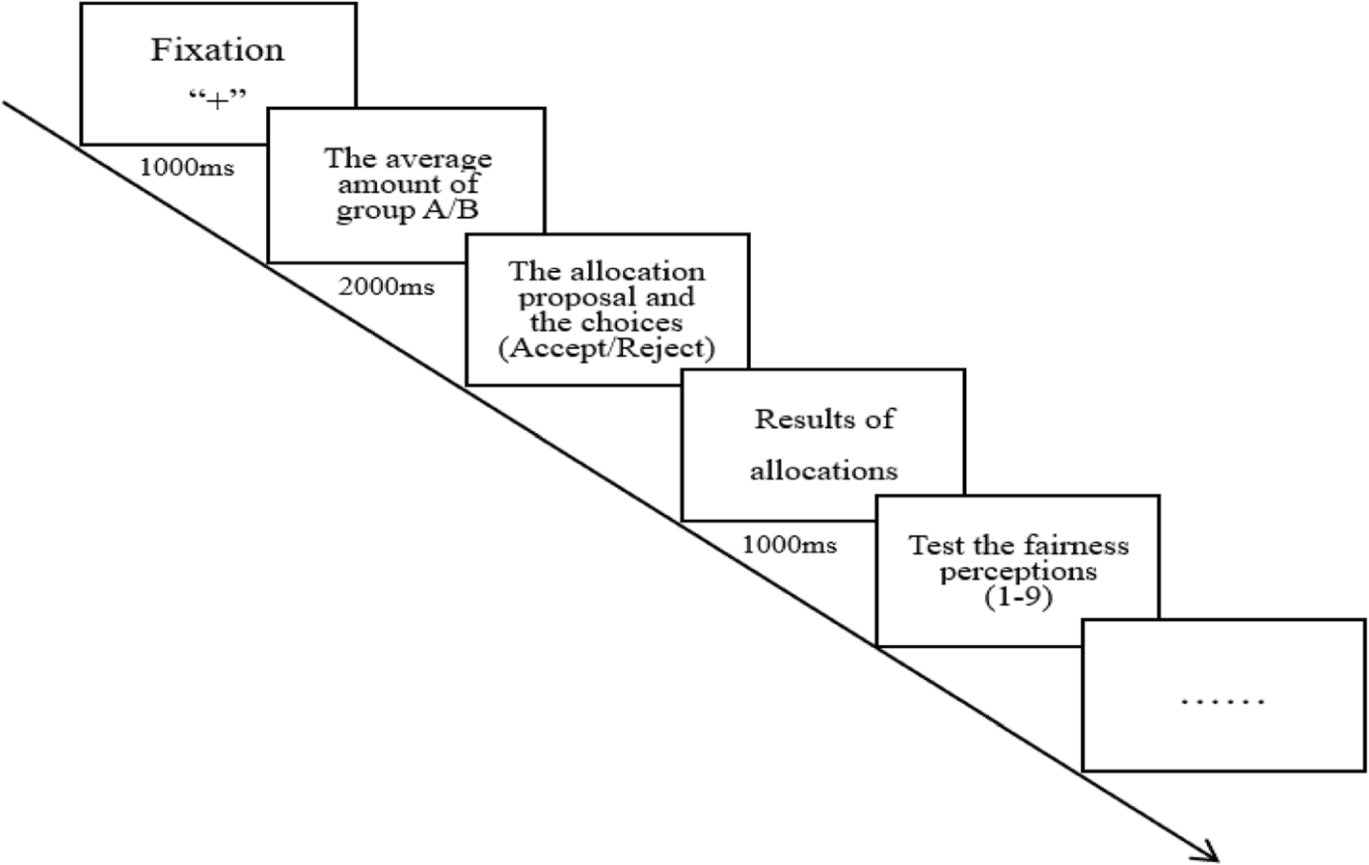
Structure of the task switching paradigm

Participants were asked to finish 5 trials for each condition, and there were 6 conditions in total (30 trials in total). Each trial began with the presentation of a situation for 1000 ms, followed by the amount of money that 7 students in group A had gained on average for 2000 ms. Then, an allocation proposal from the experimenter portraying Xiaoli would be presented (e.g., “You gain 30 yuan, he gains 70 yuan, please choose: accept or reject”) until the participant made the decision by pressing “F” to accept or pressing “J” to reject. The reaction time was recorded. Afterwards, the result of allocation (e.g., “You gained 0 yuan, and he gained 0 yuan.”) was presented for 1000 ms. Eventually, the fairness perceptions of participants were evaluated by pressing a number 1 (extremely unfair)-9 (extremely fair), which was chosen by participants according to their perception of fairness (Fig. 1).

### Experiment 2: the effect of depressive mood and social comparison on social decision-making of adolescents in a loss situation

#### Participants

In total, 208 participants (89 males) with an average age of 16.67 (SD = 0.64) were selected using cluster random sampling from a high school. We used the 13-items Beck Depression Inventory (BDI-13), Self-rating Depression Scale (SDS), and Self-rating Anxiety Scale (SAS) in a collective evaluation to select individuals, and the standards for different groups were the same as in experiment 1.

Students who volunteered to take part in this experiment were selected, and the three scales were used again before the experiment in case of a change in their mood. Ultimately, 39 normal mood students (13 males) with an average age of 16.75 (SD = 0.72) and 37 depressive mood students (13 males) with an average age of 17.16 (SD = 0.55) were recruited for the current experiment (normal mood: BDI-13 (1.92 ± 1.46), range = 4, from 0 to 4, SAS (38.43 ± 5.00), range = 24, from 25 to 49, SDS (48.88 ± 4.80), range = 24, from 29 to 53; depressive mood: BDI-13 (8.00 ± 3.14), range = 13, from 5 to 18, SAS (43.29 ± 4.48), range = 15, from 34 to 49, SDS (57.53 ± 3.74), range = 14, from 54 to 68). Again, we conducted a power analysis using G*power and the result showed that 1-β = 0.95 (effect size = 0.20, alpha = 0.05, total sample size = 76, number of groups = 2, number of measurements = 6, corr among rep measures = 0.5, nonsphericity correction ε = 1).

#### Measurements

The same scales as experiment 1 were used in the current study. The Cronbach’s alpha of BDI-13, SAS and SDS in the current study was 0.94, 0.86 and 0.83, respectively.

#### Design

The experiment design was same as experiment 1, except that the acceptance rate referred to the proportion of times participants chose to accept out of the total number of loss situations. Statistical analyses were performed using SPSS 16.0. We employed Benjamini and Hochberg’s Method to control the family wise error rate. The analyses showed that *p* < 0.053, indicating that the results were significant.

#### Procedure

The procedure of experiment 2 was the same as for experiment 1, except that the offer in the UG was a gain in experiment 1, and a loss in experiment 2.

## Results

### Results and analysis in experiment 1

It is worth noting that a preliminary experiment was conducted on 70 high school students (31 males) with an average age of 15.27 (SD = 0.479) in a senior high school in Shandong province. In this preliminary experiment it was demonstrated that 10 yuan more or 10 yuan less could alter social comparisons by the participants, caused by the differences in the experimental presentation.

#### Analysis of acceptance rates

**Fig. 2:**
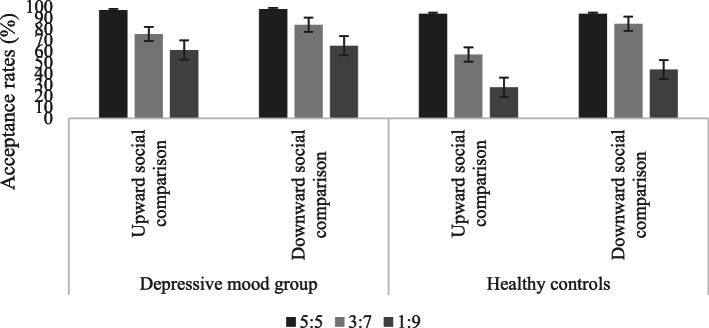
The acceptance rates of allocation proposals in the gain situation

The acceptance rates of the depressive mood group and normal mood group toward different allocation proposals in different social comparisons are shown in Table 1 and Fig. 2:

**Table 1:** The acceptance rates of allocation proposals in the gain situation (M ± SD)

Group	Social comparison	Fairness level		
		5:5	3:7	1:9
Depressive mood group	Upward comparison	96.75 ± 13.75	75.51 ± 43.47	61.08 ± 48.29
	Downward comparison	97.83 ± 10.31	83.78 ± 34.26	64.86 ± 48.39
Normal mood	Upward comparison	93.52 ± 23.39	57.05 ± 49.26	27.64 ± 44.31
	Downward comparison	93.52 ± 23.98	84.70 ± 33.05	43.52 ± 49.84

A three-factor mixed-effects ANOVA on acceptance rates indicated a significant main effect of ‘group’[*F* (1, 69) = 4.192, *p* = 0.044, *η*_*p*_^*2*^ = 0 .057], indicating higher acceptance rates of different allocation proposals in the depressive mood group (79.64 ± 4.39) than in normal mood (66.67 ± 4.57), and ‘social comparison’[*F* (1, 69) = 22.110, *p <* 0.001, *η*_*p*_^*2*^ = 0.243], indicating higher acceptance rates in downward social comparison (78.04 ± 3.19) than in upward social comparison (68.26 ± 3.48). A highly significant main effect of ‘fairness level’ [*F* (2, 68) = 38.504, *p <* 0.001, *η*_*p*_^*2*^ = 0.531] also emerged, indicating higher acceptance rates of 5:5 proposals (95.41 ± 2.21) than of 3:7 (74.77 ± 4.21) and 1:9 (49.28 ± 5.20) proposals, as well as higher acceptance rates of 3:7 proposals than of 1:9 proposals.

Additionally, significant interactions of social comparison and group were investigated [*F* (1, 69) = 5.180, *p* = 0.026, *η*_*p*_^*2*^ = 0.070]. Further simple effect analysis revealed significantly higher acceptance rates in upward social comparison for the depressive mood group (77.12 ± 4.81) than for the normal mood group (59.41 ± 5.02), whereas no significant differences were found in downward social comparison. Significant interaction of fairness level and social comparison was investigated [*F* (2, 68) = 3.660, *p* = 0.031, *η*_*p*_^*2*^ = 0.097]. Further simple effect analysis revealed that there were no significant differences of 5:5 proposals between upward and downward social comparison, while significantly higher acceptance rates of 1:9 (54.20 ± 5.83; 44.36 ± 5.52) and 3:7 proposals (84.25 ± 4.00; 65.29 ± 5.50) in downward social comparison than upward social comparison were found. Significant interactions were found for social comparison, fairness level and group [*F* (2, 68) = 3.269, *p* = 0.044, *η*_*p*_^*2*^ = 0.088]. Further simple effect analysis indicated that acceptance rates of 1:9 proposals by the depressive mood group were significantly higher than for the normal mood in upward social comparison. No other significant differences were found.

#### Analysis of fairness perceptions

**Fig. 3:**
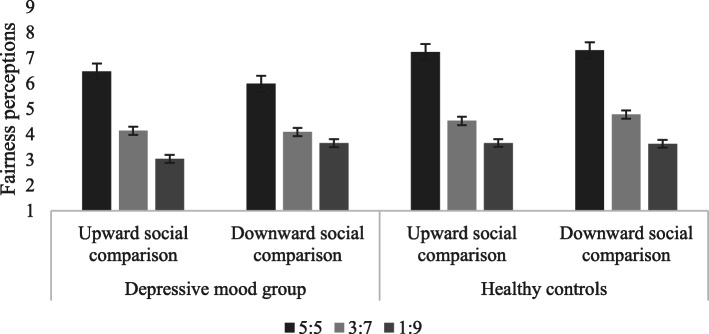
The fairness perceptions of allocation proposals in the gain situation

The fairness perceptions of the depressive mood group and the normal mood group toward different allocation proposals in different social comparisons are shown in Table 2 and Fig. 3:

**Table 2:** The fairness perceptions of allocation proposals in the gain situation (*M ± SD)*

Group	Social comparison	Fairness level		
		5:5	3:7	1:9
Depressive mood group	Upward comparison	6.45 ± 1.97	4.13 ± 1.06	3.03 ± 1.22
	Downward comparison	5.97 ± 1.95	4.08 ± 1.21	3.65 ± 1.62
Normal mood	Upward comparison	7.22 ± 1.76	4.52 ± 1.66	3.65 ± 2.45
	Downward comparison	7.29 ± 1.61	4.77 ± 1.84	3.62 ± 2.13

A three-factor mixed-effects ANOVA on fairness perceptions indicated a significant main effect of ‘group’ [*F* (1, 67) = 4.779, *p* = 0.032, *η*_*p*_^*2*^ = 0 .067], indicating higher fairness perceptions in the normal mood group (5.18 ± 0.21) than in the depressive mood group (4.56 ± 0.20). The main effect of ‘fairness level’ was also significant [*F* (2, 66) = 67.050, *p* < 0.001, *η*_*p*_^*2*^ = 0 .616], whereby fairness perceptions toward 5:5 proposals (6.74 ± 0.21) were significantly higher than 3:7 (4.38 ± 0.16) and 1:9 (3.49 ± 0.21) proposals, and fairness perceptions toward 3:7 proposals were significantly higher than 1:9 proposals. The interaction of social comparison, fairness level and group was significant [*F* (2,66) = 3.461, *p* = 0.037, *η*_*p*_^*2*^ = 0 .065]. Further simple effect analysis demonstrated that the differences in fairness perceptions toward 5:5 proposals between the depressive mood group and the normal mood group in upward social comparison were marginally significant, with higher fairness perceptions in the normal mood group. In the condition of downward social comparison, the fairness perceptions of the normal mood group were significantly higher than those of the depressive mood group. Therefore, the differences in fairness perceptions of 3:7 proposals between the normal mood and the depressive mood group were marginally significant, indicating higher perceptions of fairness in the normal mood group.

Taken together, the acceptance rates of different proposals of the depressive mood group are significantly higher than those of the normal mood group; conversely, the fairness perceptions of the depressive mood group are significantly lower than those of the normal mood group. The more unfair the proposals, the lower acceptance rates and fairness perceptions. Furthermore, the acceptance rates in downward social comparison are significantly higher than those in upward social comparison; however, there were no significant differences in fairness perceptions.

### Results and analysis in experiment 2

#### Analysis of acceptance rates

**Fig. 4:**
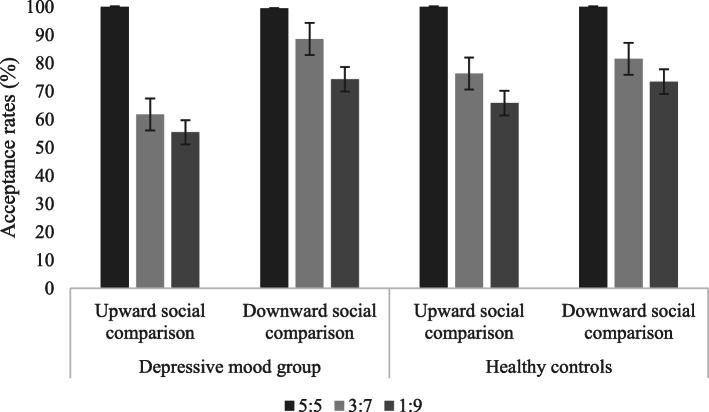
The acceptance rates of allocation proposals in the loss situation

The acceptance rates of the depressive mood group and normal mood group toward different allocation proposals in different social comparisons are shown in Table 3 and Fig. 4:

**Table 3:** The acceptance rates of allocation proposals in the loss situation (M ± SD)

Group	Social comparison	Fairness level		
		5:5	3:7	1:9
Depressive mood group	Upward comparison	100.00 ± 0.00	61.71 ± 48.11	55.42 ± 48.04
	Downward comparison	99.42 ± 3.38	88.57 ± 32.28	74.28 ± 44.34
Normal mood	Upward comparison	100.00 ± 0.00	76.31 ± 43.08	65.79 ± 48.07
	Downward comparison	100.00 ± 0.00	81.57 ± 37.01	73.42 ± 43.97

A three-factor mixed-effects ANOVA on acceptance rates demonstrated a significant main effect of ‘social comparison’ [*F* (1, 71) = 12.047, *p <* 0.001, *η*_*p*_^*2*^ = 0.145], indicating higher acceptance rates in downward social comparison (86.08 ± 2.73) than in upward social comparison (76.54 ± 3.39). A significant main effect of ‘fairness level’ also emerged [*F* (1, 69) = 22.110, *p <* 0.001, *η*_*p*_^*2*^ = 0.243], indicating higher acceptance rates of 5:5 proposals (99.86 ± 0.14) than of 3:7 (77.05 ± 4.30) and 1:9 (67.03 ± 4.65) proposals, as well as higher acceptance rates of 3:7 proposals than of 1:9 proposals. Moreover, the interaction of social comparison and group was significant [*F* (1, 71) = 4.021, *p =* 0.049, *η*_*p*_^*2*^ = 0.053]. Further simple effect analysis revealed significantly higher acceptance rates in the downward social comparison (87.43 ± 3.94) of the depressive mood group than in the upward social comparison (73.28 ± 4.89), whereas no corresponding effect was found in the normal mood group. A significant interaction of social comparison and fairness level was investigated [*F* (2, 70) = 8.187, *p <* 0.001, *η*_*p*_^*2*^ = 0.190]. Further simple effect analysis revealed that there were no significant differences of 5:5 proposals between upward and downward social comparison, while significantly higher acceptance rates of 1:9 (73.46 ± 5.19; 60.61 ± 5.63) and 3:7 (85.08 ± 4.08; 69.72 ± 5.34) proposals in downward social comparison were found compared with upward social comparison. Meanwhile, the interaction of social comparison, fairness level and group was significant [*F* (2, 70) = 3.718, *p* = 0.029, *η*_*p*_^*2*^ = 0.096], with simple effect analysis indicating that the acceptance rates of 3:7 and 1:9 proposals by the depressive mood group were significantly higher in downward social comparison than in upward social comparison.

#### Analysis of fairness perceptions

**Fig. 5:**
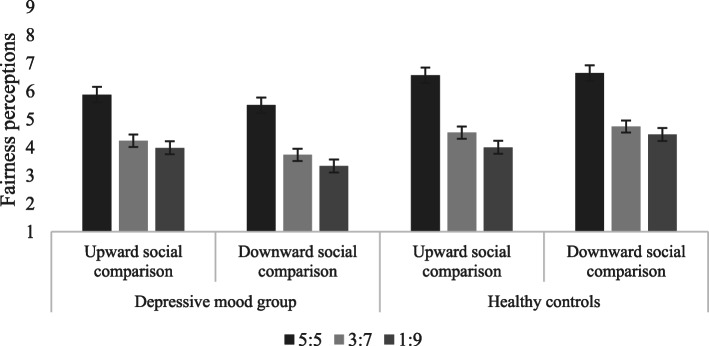
The fairness perceptions of allocation proposals in the loss situation

The fairness perceptions of the depressive mood group and the normal mood group toward different allocation proposals in different social comparisons are shown in Table 4 and Fig. 5:

**Table 4:** The fairness perceptions of allocation proposals in the gain situation (M ± SD)

Group	Social comparison	Fairness level		
		5:5	3:7	1:9
Depressive mood group	Upward comparison	5.87 ± 2.19	4.23 ± 1.87	3.98 ± 2.10
	Downward comparison	5.49 ± 2.73	3.73 ± 1.92	3.33 ± 1.84
Normal mood	Upward comparison	6.56 ± 1.94	4.52 ± 1.76	4.00 ± 1.85
	Downward comparison	6.64 ± 1.84	4.74 ± 1.83	4.54 ± 2.24

A three-factor mixed-effects ANOVA on fairness perceptions revealed a significant main effect of ‘group’ [*F* (1, 73) = 5.011, *p* = 0.028, *η*_*p*_^*2*^ = 0 .064], indicating higher fairness perceptions in the normal mood group (5.17 ± 0.23) than in the depressive mood group (4.44 ± 0.23). The main effect of ‘fairness level’ was also significant [*F* (2, 72) = 39.467, *p <* 0.001, *η*_*p*_^*2*^ = 0.523], indicating that fairness perceptions toward 5:5 proposals (6.15 ± 0.23) were significantly higher than for 3:7 (4.31 ± 0.19) and 1:9 (3.97 ± 0.18) proposals, and fairness perceptions toward 3:7 proposals were significantly higher than for 1:9 proposals. The interaction of social comparison, fairness level and group was significant [*F* (1, 73) = 6.208, *p* = 0.015, *η*_*p*_^*2*^ = 0.078]. Further simple effect analysis demonstrated that in the condition of downward social comparison, the fairness perceptions of the normal mood group were significantly higher than those of the depressive mood group. In addition, there were no significant differences in fairness perceptions between the depressive mood group and normal mood in upward social comparison.

In summary, the fairness perceptions of the normal mood group were significantly higher than those of the depressive mood group, whereas no significant differences in acceptance rates between the two groups were found. The main effect of fairness level was significant, indicating that both the acceptance rates and fairness perceptions decreased with the increase in the unfairness of the proposals. Furthermore, the acceptance rates in downward social comparison were significantly higher than those in upward social comparison, while no effect on fairness perceptions was found.

## Discussion

### The effect of depressive mood on social decision-making in adolescents

The acceptance rates of the depressive mood group, especially for the 1:9 proposals, appear to be higher than those of the normal mood group in experiment 1. Individuals with depressive mood also reported higher unfairness perceptions, replicating previous results by Harlé et al. [34]. Calvillo and Burgeno [11] indicated that rejecting unfair offers is related to intuitive thinking, whereas accepting unfair offers is related to deliberate thinking. Additionally, from the perspective of automatically negating the reciprocity hypothesis, individuals reject unfair offers intuitively but accept unfair offers reasonably and consciously, because accepting offers could contribute to the maximization of economic benefits, while rejecting them means no profits. The depressive mood group appears to have higher acceptance rates, which may be due to their “rational” mind. Another possible explanation is that depressed individuals may be more realistic about their degree of control over certain transaction outcomes: they may be less likely than the normal mood group to think that their decisions will affect either their partners or the subsequent offers they will receive, and thus may choose to accept [34]. However, some noteworthy studies have shown that the acceptance rates toward unfair proposals of depressed individuals are lower than those of normal mood [35, 55, 61]. Different results might be due to the different degrees of depression in participants. Harlé et al. [34] selected nonclinically depressed patients with less severe depression, which is similar to our depressive mood group, whereas most other studies selected clinically depressed patients with more severe depression [63]. Wang et al. [68] reasoned that there might be a significant negative correlation between the acceptance rates of depressed individuals and their clinical severity, which was confirmed in the current study based on individuals with less severe depression. Taken together, these findings indicate that depressive mood has an important effect on the social decision-making of adolescents, which is in line with H1.

However, the results of experiment 2 showed no differences between the depressive mood group and the normal mood group, which was different from experiment 1. Meanwhile, the acceptance rates were both relatively higher in the two situations. That is, healthy individuals showed higher acceptance rates toward unfair proposals in the loss condition compared with the gain condition, a result consistent with previous reports [44]. Studies have demonstrated that in different situations, the nature of the situation can affect the interpretation of the task, thus changing the tendency of game [5, 20]. According to prospect theory, individuals are particularly sensitive to loss when making behavioral decisions; specifically, the pain of loss is greater than the pleasure experienced for the same level of gain [40, 60]. Moreover, compared with adults, adolescents concentrate more on the outcomes when making decisions [59]. Therefore, they may avoid loss as much as possible in loss situations, showing increased acceptance of unfair proposals. Such an outcome may indicate that adolescents in different situations make decisions in different ways, and the situation plays quite an important role in social decision-making.

### The effect of fairness level on the social decisions of adolescents

The main effect of fairness level in the two experiments was found to be significant. Consistent with available studies [49, 83], the results of the current study show that within the loss or gain domain, the acceptance rates and fairness perceptions of participants both decline with an increase in the unfairness level of proposals. According to equity theory [23], the reason for the rejection of responders might be explained by the aversion to unfair proposals. Our findings are supported by other reports that have found that individuals may experience aversion when facing unequal outcomes [13]. Furthermore, the stronger the negative emotional experience triggered by unfair proposals, the lower the acceptance rate of the responders [65]. In contrast, fairness at a higher level may heighten the fairness perceptions of participants, which could lead to higher acceptance rates of proposals [44]. Meanwhile, Güroğlu et al. [33] reported on the developmental changes in fairness considerations for individuals entering adolescence, demonstrating the preference by older adolescents for fair allocations. Consistent with H2, this result suggests that the fairness level of proposals might have a significant impact on the social decision-making of adolescents, which is supported by previous literature.

### The effect of social comparison on social decision-making in adolescents

The results also indicated that the acceptance rates were significantly higher for downward social comparison than for upward social comparison. Compared with upward social comparison, the acceptance rates of 3:7 and 1:9 proposals were significantly higher for downward social comparison. It is reasonable to question whether fairness perceptions were heightened by downward social comparison. Analysis of the two experiments did not find more fairness perceptions in downward social comparison. According to Smith et al. [56], through social comparison, the perceived disadvantage viewed as unfair is likely to result in anger and dissatisfaction. Even if the consequences are “objectively” profitable for individuals, unfavorable social comparisons could lead to resentment [10, 62], which could further increase the level of depression and anxiety in individuals [42]. By contrast, participants could experience stronger positive feelings in downward social comparison [25]. Thus, it can be pointed out that the negative feelings caused by social comparison might influence social decision-making, especially for adolescents who are sensitive, unstable and have poor self-control.

In addition, it has previously been suggested that upward social comparison could be threatening for individuals’ self-concepts [52], whereas downward social comparison may elicit positive self-assessments [69] and more cooperative behaviors [74]. Moreover, Xie and Lu [71] reviewed previous literature and proposed the corresponding relationship between social reference point and the condition of social comparison, indicating that social comparison might have an effect on decision-making by influencing individuals’ self-concepts, emotions and recognition of fairness, while its specific pathway depends on the particular decision-making situation. Consequently, we reasoned that in the UG, the effect of social comparison on individuals’ emotions and self-concepts might have a further impact on decision-making.

### The interactive effect of depressive mood, social comparison and fairness level on social decision-making in adolescents

Notably, the comparison of acceptance rates between the two experiments showed a significant interaction of social comparison, fairness level and group. In the gain situation, the acceptance rates of individuals with depressive mood were significantly higher than those of the normal mood group in upward social comparison. Individuals with depressive mood did not show higher rejection rates, but made decisions based on the principle of profit maximization. Such decision-making may be due to the negative self-assessment of individuals with depressive mood [3]. Specifically, individuals with depressive mood assume that it is normal for them to receive less than others when allocating funds; thus, they did not show higher rejection rates in upward comparison. However, when facing 3:7 and 1:9 proposals in the loss situation, the depressive mood group showed significantly higher acceptance rates in downward social comparison than in upward social comparison. According to the social reference point proposed by Xie and Lu [71], upward social comparisons correspond to social losses, while downward social comparisons correspond to social gains. Thus, upward social comparisons in loss situations might imply “double loss” [45], which may result in participants’ vigilance against double loss and further lead to higher acceptance rates in downward social comparison in loss situations. Meanwhile, research on patients with depression demonstrated that loss aversion was higher in patients with depression than in the normal mood group [12], which may be a result of the different decision-making styles between individuals with depressive mood and healthy individuals in different situations and different social comparisons.

Moreover, analysis of fairness perceptions in the gain situation indicated significant interactions of social comparison, proposal type and group. This tendency probably occurs because individuals with depressive mood tend to interpret stimuli selectively in the environment and interpret neutral or ambiguous stimuli as negative or less positive [68]. Indeed, major depression is associated with a negative emotional bias. Compared to the normal mood group, depressed patients judge emotional stimuli too negatively [55] and experience more negative feelings. The analysis of fairness perceptions in the loss situation found no significant interactions of social comparison, fairness level and group. In fact, available research has demonstrated that individuals are more sensitive to loss situations [9], and individuals with and without depression both show a consistent high sensitivity to unfairness in loss situations [64], which could further lead to a “floor effect” for fairness perceptions.

To conclude, as was predicted, we found that the interaction of social comparison, proposal type and group was significant for acceptance rates in the loss situation, whereas in the gain situation, the interaction was significant for acceptance rates and fairness perceptions.

### Theoretical and clinical significance

#### Theoretical significance

The present study has explored the influence of the level of fairness on teenagers’ social decision-making, finding that adolescents have a higher acceptance rate and fairness perception of fair proposals than unfair proposals, which indicates that fairness plays an important role in teenagers’ social decision-making. This finding enriches the research on fairness theory and its related areas. The current study has also discussed the influence of social comparison on teenagers’ social decision-making, thereby enriching the theory of social comparison. In addition, this study has examined the social decision-making of adolescents under the framework of gain and loss. It has broadened the applicable range of the framework theory from risky decision making to social decision making. Most importantly, this study has focused on depressive mood in adolescents, which is more common than depression. It has examined the influence of depressive mood on social decision-making, highlighting a potential indicator for the preliminary diagnosis of depression.

#### Clinical significance

The current study found that there are differences in the fairness perception and acceptance rate between depressive mood and normal mood individuals in social decision-making, which may provide an indicator for the preliminary diagnosis of adolescent depression. Similarly, this effect could also be used as a means to judge the therapeutic effect of patients with major depression. By comparing the differences in judgment and perception of unfair events between depressed individuals and normal individuals, it may be possible to judge whether individuals have depression or not, even when concealed by masking behavior. Wang et al. [68] reasoned that there might be a significant negative correlation between the acceptance rates of depressed individuals and their clinical severity, a possibility which would support the reliability of this method for detecting depression.

### Limitations and strengths

This exploratory study investigated the effect of depressive mood and social comparison on social decision-making among adolescents in the context of gain and loss. Several specific findings emerged, with implications for future psychosocial education. First of all, the social decision-making of adolescents is particularly affected by social comparisons, implying that negative social comparisons could negatively impact individuals’ physical and mental development. To date, many studies have begun to emphasize the role of social comparison. For instance, Lu et al. [47] demonstrated that insecurity occurs when individuals are faced with adverse social comparison results, while Cotier and Toulopoulou [15] further emphasized that negative social comparison appears to be a potential sign of depression. Thus, helping adolescents acquire appropriate social comparison methods could not only be conducive to their social decision-making, but also benefit their physical and mental health. Second, despite the fact that depressive mood does not meet the diagnostic criteria for depression, it could negatively impact on social decision-making by adolescents, suggesting that we should pay more attention to the prevention of adolescent depression. Third, the interaction of social comparison, depressive mood and proposal type demonstrates that besides emotional state, cognitive biases and social factors could also have an effect on social decision-making. Behavioral decision boosting may therefore represent an avenue for appropriate interventions and for providing guidance to adolescents on their social decision making.

This study had several limitations, opening up the potential for related future research. First, we only assessed the social decision-making of individuals with depressive mood rather than clinical patients. According to Wang et al. [68], there may be differences in individuals with different severities of depression, indicating that future research is needed to evaluate the differences in acceptance rates in individuals with different severities of depression. Male participants were also underrepresented in the current study, highlighting the need to expand the sample size further to gain a more nuanced understanding of the impact of depressive mood and social comparison on social decision-making among adolescents. Another limitation was the lack of analysis on reaction time. As reported, delayed decision-making could reduce unfair rejection rates [50], suggesting that future studies should examine the impact of reaction time on social decision-making. Importantly, although the study avoided the extra cognitive resource consumption caused by both the distinguishing of similar experimental procedures and the practice effect, the influence of individual differences could not be controlled in this study. Future studies may also investigate the effect of situations on social decision-making using a within-subject design. The other noteworthy point is that factors such as personality [6], empathy, justice sensitivity [76], value orientation and social distance [77] can also affect social decision-making. Future studies may benefit from exploring the relationship between these factors and social decision-making. In addition, the present study found that individuals with depressive mood have different social decision-making behaviors in different situations from a behavioral perspective. Future studies might provide more detailed information about the neural substrates of this behavior by availing of neuro-imaging techniques.

## Conclusions

The conclusions of this study are as follows:

1. When faced with fairer allocation proposals, individuals have higher fairness perceptions and show higher acceptance rates of the allocation proposals;
2. The acceptance rates in downward social comparison are higher than those in upward social comparison, whereas no differences in fairness perceptions between the two social comparisons were found;
3. In the gain situation, the depressive mood group showed higher acceptance rates than the normal mood group, while no differences were found between the two groups in the loss situation. However, the depressive mood group showed higher unfairness perceptions in both situations;
4. An interaction between depressive mood, social comparison and proposal type was found. Specifically, individuals with depressive mood showed higher acceptance rates and more unfairness perceptions than the normal mood group when faced with upward social comparison in the gain situation.

## Data Availability

The datasets during and/or analyzed during the current study are available from the corresponding author on reasonable request.
